# Answer to Dr. Drake’s Interrogatories

**Published:** 1853-10

**Authors:** 


### DENTAL REGISTER OF THE WEST.

VOL. VII. OCTOBER, 1853. No. 1.

### Original Communications.

### *Answer to* DR. DRAKE'S *Interrogatories—Continued.*

The next question to be considercd is, "What is the effect of repeated salivations on the teeth and gums?" This is an important question, and particularly so, as a large majority of teeth affected with caries are supposed to be so by the use of mercuiial remedies. The practitioner of Dental Surgery should be able to decide if this so oft repeated assertion is true or false—"My teeth have been ruined by Calomel." Numerous cases like the following present themselves in our practice: An individual calls with painful tooth-ache—his teeth have never ached before, he has just been sick, his physician has likely given him a dose of calomel. In a few days he is about, and the first exposure, in his relaxed and susceptible condition, tooth-ache sets in. A few days' suffering drives him to the Dentist, when lo, and behold! half of the teeth are decayed, and as they had never ached before, and really never been examined, he thinks they have all decayed since the dose of calomel—and this is the scape-goat which must carry off all the blame. The remedy it is supposed has really eaten, as it were, in this short time, holes in the teeth. We will examine this question Chemically and Pathologically. The class of cases illustrated above represents a large majority of those which are presented, and if true, must be the result of direct chemical action. Can this be so? The proto chloride or sub muriate of mercury contains to the 100 parts mercury 13.99 muriatic acid, and 4·16 oxygen. The mercury very largely forming the base, which in its pure state is tasteless, and has no effect whatever on the teeth. It is therefore only in its union with oxygen and some acid, that a chemical action could be expected, and unless that acid should have a greater affinity for lime than the phosphoric, and the latter more affinity for the mercury than the former, no such action could take place. This we find to be directly the reverse, and so according to the laws of chemical affinity, no action could take place, and experiment fully demonstrates this law of chemical science; for teeth immersed for months in this article show no signs of decomposition.

If this was not so, we should expect a much greater injury from the use of common table-salt. The muriate of soda—here we have 46 parts of acid to 54 of soda—a much more soluble article in the mouth, and when dissolved a much larger amount of acid brought in contact with the teeth. But the same individuals, who so loudly denounce calomel as injurious to the teeth, will actually use salt as a dentrifice.

Whatever injury, therefore, may result to the teeth from the administration of mercurial remedies must be looked for in some other way than by direct chemical action, and those numerous cases of tooth-ache must be accounted for in some other way. The most reasonable conclusion is, that these teeth have been decaying for some time, but during the healthy operation of the economy, no just cause for derangement has occurred, and hence no pain—but the indisposition which rendered it necessary to take any medicine, has vitiated the secretions of the mouth. They become irritants, and by the time health is restored to the general system the teeth are in a fit state to be affected by the slightest exposure, and hence we have tooth-ache. The teeth now decay rapidly and this condition is of so frequent occurrence after an attack of disease that it goes far to establish the position we have taken in our reply in answer to the question "What is the cause of decay?"

The question now comes up, does that condition of the saliva which exists during salivation, iuduce decay of the teeth?" To determine this satisfactorily, we must examine this secretion when in this condition, and see if the change is of such a character as to give the power of decomposing the teeth. We have repeated experiments made on saliva from the mouths of salivated persons, and these have been made by our most celebrated chemists. These experiments have been made to test the changes in this fluid, and if possible detcct the presence of mercury. The fact whether mercury exists or not in this fluid can have but little influence, however, in its action on the teeth. Changes, however, which wonld destroy its alkaline property, rendering it acrid or of an acid character, would have an important bearing on this subject.

Simon in one case in a specimen of saliva from a patient who had been laboring under salivation for two or three weeks, observed an acid re-action from the presence of free acetic acid. The saliva contained a very large amount of semi-fluid fat and albumen, and under the microscope showed an immense number of fat vessicles and some epitheliem cells. His analyses gave the following result:

| | |
|---|---|
| One thousand parts of saliva gave water | 974.12 |
| Solid constituents | 25.88 |
| Yellow viscid fat | 6.94 |
| Plyalin with extractive matter and traces of casein | 3.60 |
| Alcohol extracts with salts | 7.57 |
| Albumen | 7.77 |

L. Heretier gives the mean of three analyses of this fluid during mercurial Ptyalism—and thus showed—

| | |
|---|---|
| Water | 970.0 |
| Organic matter | 28.6 |
| Inorganic matter | 1.4 |

The same individual has given us the mean of ten analyses of normal saliva, which gives the following:

| | |
|---|---|
| Water - - - - - - - | 986.5 |
| Organic matter - - - - - - | 12.6 |
| Inorganic matter . - - - - - | 5 |

This diminution of water as the principle constituent of saliva does not always take place during salivation, but depends somewhat on the stage of the affection—thence Dr. Wright gives us an analyses of saliva from a patient laboring under mercurial Pytalism—it contained of water 988.7.

Gmelin and Thompson, and many others, have given careful analyses of this fluid, and show much the same changes as those already alluded to. We find but seldom indeed that the saliva, during salivation, exhibits an acid reaction, and although the changes are such as to warrant the belief that teeth already decaying may be accelerated by this condition of the secretions of the mouth, much in the same manner as when they are vitiated from any other cause, yet the teeth being sound would not necessarily be injured thereby more than from any other derangement of the healthy condition of the mouth.

We are aware that in expressing this opinion we differ from many of our brethren in the profession, yet when we see many who have been badly salivated with perfect dentures—and this often repeated two or three times—we are led to enquire farther into the cause of decay and generally meet with other and satistactory reasons.

The remarks we have made have special reference to the effect of salivation after the teeth are matured, and the chemical action which we may expect from this medicine itself or through the changes it affects on the seretions in which the teeth are constantly placed.

In our reply to the queston "What is the nature of that diathesis or constitutional predisposition or disorder (if any) which so often occasions decay in the deciduous teeth of our children?" and "To what cause, external or pathological, local or constitutional, shall we ascribe the premature decay of the second teeth in the West?" We pointed out the effect of diseased action on the developement of these organs, and contended that an organ to carry out a perfect developement, must be healthy in its operation. That a departure from a healthy function must prevent a perfect assimilation of the parts being formed. The reasons then assigned have a special bearing on this question, and render but little necessary to be said in relation to the injurious effect of salivation during the formation process of these organs. The mucous membrane as well as the glands which supply the saliva is much excited during the action of this medicine, and all the regular and healthy functions connected with this important tissue must be disturbed.

The injury to the gums from repeated salivation is a matter about which there cannot be much doubt. In salivation we have inflammation and swelling of the gums, and this inflammation is not confined to the gum itself, but extends to the periostium of the teeth, hence this membrane becomes thickened or enlarged and the teeth are raised in the socket and loosened; there is, therefore the same condition existing to some extent which we find in inflammation of this membrane from any other cause. This irritable or inflamed condition of the periostium and contiguous parts generally lead to some of the following results:—seperation of the gums from the necks of the teeth—thickening of the alveola-dental membrane—absorption of the alveolar processes, and with this wasting of the gums. These conditions, and the injury resulting therefrom, will depend much upon the duration of salivation and the frequency of its operation, also, the constitutional habit of the body.

Take, for instance, an individual of strumous, cachetic, or scorbutic habit, and severe salivation would often result in necroses and exfoliation of the alveolar processes, and this appears to be particularly the case if the system is tainted by syphilitic disease. In all conditions of the system, where we have a tendency to soft and spongy gums, with accumulations of salivary calculi, repeated salivation is excessively injurious.

The teeth becoming loose appear never to regain their firmness. The processes are gradually absorbed, the gums wasted away, and the teeth drop out. This occurs, too, most frequently, without any special decay. True, when the necks of the teeth become exposed, and the secretions of the mouth, from some cause or other, become acrid, the teeth soften and decompose at this part. This is often the case when the agent inducing decay is too weak to act upon the enamel.

From long observation, and the condition which the secretions of the mouth present under chemical analyses, we are led to believe that mercurial salivation may injure the dental organs during their developement, and is always more or less injurious to the gums. But that no direct chemical action from this article is produced upon the teeth after they are matured.

The following remarks from Prof. Bond's "Dental Medicine," embrace, as we think, very correct views, and we fear describe too truly the course which many pursue, instead of combatting a very erroneous and unreasonable opinion.

He remarks that, "there is no reason to believe that the use of mercury is injurious to the teeth, when salivation is not induced; yet caries of these organs is very often attributed to it. People are exceedingly apt to confound the post hoc with the propter hoc; and dentists are as liable as other men to fall into the error. A patient who has escaped a severe attack of fever, finds his teeth rapidly decaying; in great alarm he applies to the dentist. The latter glances at the mouth and with a look of boding sagacity, inquires if the patient has been taking calomel. The patient replies that he has been taking more or less, and then the man of science, as he is presumed to be, launches forth for the hundreth time into a bitter diatribe against mercury as the origin of all.

And why may not the lamented caries be as justly charged upon the tartar emetic or magnesia, which the patient may have taken simultaneously with the calomel? or why does not the dentist seek for all the sufficient cause of devastation in the acid saliva of a fevered mouth for weeks consecutively? Why transfer the blame to the remedy by which the fever was subdued, and cast implied and serious censure upon the physician, whose judicious employment of the villified drug has, perhaps, saved the patient's life?

Until we have other information than we now possess, we cannot believe that the proper employment of mercury is injurious, and while we reprobate its abuse and would think the physician unpardonable, who would be careless or reckless in the use of a medicine capable of doing so much harm, we cannot but regard that man as the author of greater evils, who by silly declamation against an important remedy, fetters the physician in his contest with 'the most formidable diseases.'

What are the effects of Tobacco on the teeth, and are those of chewing and smoking the same?

The following answer to this question has been handed us by Dr. Berry, one of the committee appointed by the society to answer the interrogatories of Dr. Drake. It it will be observed that it is taken for granted that the tobacco is in its pure state, and not so injured by the addition of deleterious agents in its manufacture as to render a different action on the teeth possible. While the friction to which Dr. Berry alluded often does abrade the teeth—yet we have no doubt this is one cause of its preservative effects on the teeth. Teeth used most in the mastication of our food are generally most sound, and this is not always because we select those which are best for such use, for there was a time when there was no apparent difference, but those used have, by the friction, been kept clean, while the other side either accumulates tartar, or the teeth decay. The sedative and narcotic effect of tobacco certainly allays the irritable condition of careous teeth, thus preventing tooth-ache, &c. Any agent capable of this and not a decomposing agent of the teeth must certainly in such circumstances, be preservative; if not, inflammation of the dentine cannot facilitate decay. Inflammatory diseases produce an acid condition of the secretions of the mouth—this vitiated state of the secretions induces tenderness and inflammation of the dentine—here is a condition of decay which we believe the use of tobacco will, by changing the nature of the secretions, and subduing the irritable condition of the caries, prove beneficial.

The benefit of friction may be obtained by the chewing of wood, sole-leather, or something even more tough and cheap, but the sedative and narcotic agent would be wanting, and although, with Dr. Berry, we are utterly opposed to the "use of the weed," yet we know not any other narcotic and sedative which a majority of our gentlemen patients would so pertinaciously and readily use.

We attribute the relief of tooth-ache, by smoking, to the sedative effect of tobacco thus used :—

A knowledge of the chemical constituents of tobacco does not enable us to answer the question respecting its effects on the teeth. From its active poisonous properties it might be inferred, a priori, that its habitual use must be injurious to the system generally. We do not doubt that such is the fact. If so, it follows that, indirectly at least, it is prejudicial to the dental organs. But its direct effect on them can only be learned by observation and experience.

The friction from chewing tobacco has a tendency to abrade the teeth, and we sometimes meet with cases in which they are worn away by it nearly or quite to the gums; while those who chew it only on one side of the mouth are occasionally seen with one side of the face shorter than the other.

Although opposed to the use of tobacco in all its forms, we believe that when chewed it often exerts a preservative influence against decay of the teeth. We think this the case usually, except with persons whose constitutional diathesis and defective organization of the teeth predispose these organs to be attacked by caries. The teeth of those who chew tobacco are generally less liable to become carious, and when invaded by this disease its advances are slower than with those who do not use the weed. Teeth in which fillings are poorly inserted, or in which cavities well filled extend to their necks below the edge of the enamel, are, from the adjuvant of tobacco chewing, more secure from the danger of a renewal of decay. These results are produced in part, probably, from the more frequent removal of the fluids and foreign matter about the teeth, but principally from an antiseptic influence of tobacco.

The sedative and narcotic effects of tobacco in allaying tenderness and pain of the teeth are too well known to require any remark respecting them.

Although the gums sometimes receive detriment from long continued pressure of the quid, and with some constitutions its sitmulant and narcotic action may enfeeble them, yet *caeteris paribus* they are seldom found more diseased in the mouths of tobacco chewers than in those of others.

The coloring matter of tobacco, when it is chewed for a considerable length of time, generally stains the teeth, presenting a disgusting appearance.

The effects on the teeth of chewing and smoking tobacco differ, as the latter exerts no mechanical action on them. The empyreumatic oil from smoking often collects on the teeth extensively, but it probably has little or no agency in inducing or preventing caries. Smoking is doubtless more prejudicial to the gums than chewing, as it has a more debilitating influence on them. And since a normal condition of the dental sockets is in a great degree dependant on a healthy state of the gums, probably the dental apparatus in many instances suffers serious injury from the habit of smoking tobacco.

Has the tartar of the teeth a constitutional origin? There is no subject in connection with Dental Surgery on which a greater variety of opinion exists. M. Delabarre, a celebrated French author, contends that it is an " exhalation from the mucous membrane of the gums," that this membrane when in a state of irritation, throws off a "salino-terreous exhalation," and that this attaches itself to some foreign substance, which acts as a nuceleus for the deposition of the tartar. He institutes a comparison in relation to the formation of urinary calculi, and that in long continued gout there is a deposition of "calcareous" matter in the sinovial fluid of the joints affected by this disease. This theory, in relation to the formation of salivary calculus, would depend somewhat on the irritation existing in the mouth, and that when the gums are most inflamed the tartar would accumulate most. This, although to some extent true, yet the tartar is really the irritant, and is the cause of the inflammation. Irritation does not always increase the deposition of tartar and we do not always find it most located when the most inflammation exists in the mouth. Jourdain thinks that the periostium of the teeth have glands which secrete the tartar.

Serres says that the mucous membrane has certain glands "whose particular function it is to secrete this substance." M. Mandl says "tartar is composed of infusoria," "having their origin in the vitiated mucus which is always mixed with it." This latter theory has been of late somewhat revived, and a soap recommended for destroying the animalculæ. There can be no doubt that infusoria has, with the microscope, been detected in the decomposed and softened particles of food and mucus which are suffered to remain for any length of time about the teeth, and these may be ultimately attached to and mingled with the soft brownish or white tartar which is frequently found about the teeth. Such appear to be rather extraneous to the tartar itself, and are not necessary to it. The mucus secretions we should always expect to find in this substance—because of the intimate association of the mucous membrane and the viscid condition of its secretion where this substance is much deposited. A very prevalent opinion with many is that the tartar is an encrustation of vegetable and animal matters left about the teeth after eating. This opinion has been entertained in part from the fact that those who are generally troubled most with this accumulation are negligent about cleaning their teeth. This negligence, however, does not always give as a result an accumulation of this substance, so that we have very often freedom from tartar as well as decay where no possible attention is paid to the teeth.

Your question, then, is important and pertinent: "Has the tartar of the teeth a constitutional origin?" If so, its physical characteristics would form, to some extent, a diagnosis, of the temperament of the individual and the general state of health.

We have three marked and distinct varieties of this substance; these, although different in general appearance, yet possess, but in different quantities, the same elements.

We have first a hard, brittle variety, which adheres very closely to the teeth—insinuates itself under the free margin of the gums, and scarcely ever collects in very large quantity.

We then have a second variety, of a dark, or yellowish brown color, not near so hard as the former, and usually collecting in rather large quantity. This, although insinuating itself under the edge of the gum, yet it often almost covers the teeth, and this is especially the case when it partakes somewhat of the characteristics, at least in color and softness, of the third variety, which is almost white and so soft as to be broken up between the fingers. These three varieties are often more or less blended with each other; sometimes the black is dark brown, sometimes the brown is very light, and sometimes the white approaches the light brown. These modfications appear to result from the effect of disease, the prevailing constitutional diathesis and the habit of cleanliness. An individual in whose mouth, for instance, the dark or yellowish brown would naturally collect, under ordinary care in cleaning the teeth; yet, if this is neglected, more of the viscid mucus is mixed up and combines with the tartar, rendering it of a lighter color and more soft—all the different changes which are observable in the appearance of this substance we think can be thus readily accounted for: In the chemical analyses of this substance we find a great variation in the results obtained, but really not more than its physicial characteristics would indicate. Berzelius gives us the following as the result of his analyses:

In one hundred parts he found Phosphate of lime

| | |
|---|---|
| and Magnesia - - - - - - - | 79.0 |
| Salivary mucus and Salivarine - - - | 13.5 |
| Animal matter - - - - - - - - | 7.5 |
| | 100.0 |

Dr. Duinelle gives the following as the result of his anlyses :

| | |
|---|---|
| Phosphate of lime - · - - - - | 60 |
| Carbonate of lime - - - - - | 14 |
| Animal matter and mucus - - - | 16 |
| Water and loss - - - - - - | 10 |
| | 100 |

In these different analyses we are not informed of the variety of tartar used in each case. The fact, however, is generally known that the dark and hard contain more of the earthy elements than the soft, and in proportion as this substance is soft and approaching white do we find the mucus and animal matter increased.

This substance of late has most generally been termed by Dental authors, Salivary Calculus, from the fact that it is suppossd to be a product from this fluid. That this is probable may be inferred from the fact that the saliva by analyses gives nearly if not all of the elements. Dr. Wright, as the result of his analyses of this secretion in its healthy state, gives the following:

| | |
|---|---|
| Water - - - - - - - | 988.1 |
| Plyalin - - - - - - - - - | 1.8 |
| Fatty acid - - - - - - - - | .5 |
| Chlorides of soda and Potassium - - - | 1.4 |
| Albumen with soda - - - - - - | .9 |
| Phosphate of lime - - - - - - - | .6 |
| Albumenate of soda - - - - - - | .8 |
| Lactates of soda and potash - - - - | .8 |
| Sulphocyanide of Potassium - - - - | 9 |
| Soda - - - - - - - - - - | .5 |
| Mucus with ptyalin - - - - - - | 2.6 |

Dr. Wright gives as the result of his analyses of three or four different forms of morbid saliva, the following proportions of lime and mucus, which we give as in part the elements of the tartar.

| | |
|---|---|
| In Fatty saliva he gives of Phosphate of lime | 1.8 |
| Mucus | 2.4 |
| In sweet saliva mucus with a trace of ptyalin | 2.6 |
| Phosphate of lime | 1.9 |
| In Bilious saliva | |
| Mucus | 1.6 |
| Phosphate of lime | 2.3 |

In all these analyses different proportions of animal matter is given yet sufficient to enter into the composition of the tartar. We have given these analyses to show that the elements for the production of this substance is found in the saliva in a healthy as well as a morbid state. That it is primarily obtained from this fluid we think is conclusive from the following reasons. First, it has been found in the ducts which pour out the saliva—here it could not be formed from the decomposed aliment which is suffered to remain about the teeth—nor from the glands which Jourdain thinks is "scattered over the periosteum of the teeth," and which secrete the tartar.

*Second*, the largest amount of tartar, is usually collected on the teeth opposite the salvary ducts, on the labial face of the superior molars, and the lingual face of the lower incisors. This fact favors the idea, that after the saliva has been thoroughly mixed up with the mucous secretions, and foreign substances of the mouth, it does not so readily deposit this substance. And this is particularly the case when there is not a marked pre-disposition to accumulations of this kind.

M. Delabarre's theory, that it is an exhalation from the mucous membrane of the mouth when in a state of inflammation is not by any means tenable; for as he also remarks, that the black dry tartar which is deposited around the necks of the teeth of such only as are possessed of good constitutions, is never in large quantities—is dissolved in muriatic acid with difficulty, while the dry yellow tartar found upon the teeths of bilious persons, dissolves more readily in it; but the soft white tartarfound upon the teeth of individuals of mucous temperaments, he tells us, is scarcely at all soluble in the acids, but "is readily dissolved in the alkalies."

It is a well known fact, that the mucous membrane of the mouth during inflammation, throws out an acid secretion, and some of the worst cases of inflammation, or even long continued irritation of this membrane, gives us no deposition of tartar. The extraneous matter which collects about the teeth during this time, appears to be only a mixture of mucus and decomposed food, which merely glutinates together and never acquires enough of the earthy elements to be called tartar.

If M. Delabarre's theory was correct, no other kind of tartar could be formed during inflammation of this membrane when it produces an acid state of the secretions, but the white soft variety, and this is found only in the mouths of those of mucous temperaments. We think he is here in error, and that the acid ståte of the secretions of the mouth, almost entirely precludes the formation of tartar, while this condition exists. M. Delabarre's theory would preclude the formation of the soft white tartar, when the secretions of the mouth were alkaline; for he remarks, it "is readily dissolved in the alkalies." An acid state of the secretions of the mouth induces and keeps up inflammation of the mucous tissue, and is one great cause of decay of the dental organs, and observation confirms the fact, that teeth much disposed to accumalations of tartar, are not much subject to decay, and carious teeth filled with a deposition of hard tartar, is supposed to be in a tolerbly preserved state. The presence of tartar around the necks of the teeth, in contaet with the gums, is an irritant, just as any other foreign substance would be, and hence, we have inflammation of the gums, resulting from the presence of tartar.

If tartar was a deposit from the mucous secretions, it would be just as apt, or more so, to first collect about the teeth farthest off the openings of the salivary ducts. The reverse is true, yet the fact is well established, that when the predisposition exists, and there is an irritable condition of one side of the mouth, so that the teeth are not kept clean, and are not used for mastication, tartar will collect about them. This however, does not prove that it would not collect about the other teeth in like manner, if not used, but it proves that the friction of the food in mastication, prevents its collection on the teeth used, and also, that the use of the teeth is essential to their preservation and the health of the gums.

What are the constitutional temparaments most disposed to accumalations of salivary calculus?

There is a peculiar trait about every individual, and every-system appears to be characteristically marked with an idiot syncracy of its own. These have by long continued observation become indexes by which we are guided in our diagnosis of habit of body, predisposition to disease, &c. There are however, extraneous and accidental circumstances, modifying and changing somewhat these almost fixed laws of the animal economy. There are also by marriages and inter-marriages, mixing up and blending of these peculiar traits or temperaments, which modify also disease, as well as the function of the different organs.

A most perfect development of constitution, giving firm, rosy and healthy gums, with a good denture, but seldom exhibit much collection of salivary calculus. The small quantity which is sometimes found in the mouths of such, when in health, is generally deposited on the lingual surface of the inferior incisors opposite the ducts of the sub-maxillary glands. In such cases, the tartar will be found dark and hard, such constitutions—and they are generally such as where the sanguinous temparament predominates, with firm muscle and healthy mucous tissue, exhibiting rosy colored gums and lips. If in such cases, large collections of salivary calculus is found, it will be found to result from protracted indisposition, the result of excess of living, inducing debility of the muscular fibre, and an altered condition of the firm red-roseated appearance of the mucous membrane. The healthful circulation of the system, which proclaimed its perfect vitality and vigorous condition, through the delicate mucous membrane, has become exhausted, and we may now, through a vitiated condition of the secretions of the mouth, expect disease of the dental organs, accumalations of salivary calculus, and irritable and spongy gums. This irritable condition of the gums, extends from tooth to tooth around the mouth, as the deposition of tartar becomes manifest, under the free margin of the gums. We may generally expect, as the mucous tissue becomes involved in the disease, its viscid secretions &c., will be mixed with the tartar itself; thus, diminishing the relative amount of its earthy constituents, and increasing that of the mucus. The color also is affected by this, the tartar losing its black or dark brown appearance—and assimilating to the yellow brown. This latter, however, Dr. Harris remarks, is generally found in the mouths of those of "bilious temperaments, at least where this predominates," which we think, in ordinary cases, uninfluenced by the conditions of the secretions and health alluded to in the former case, is correct. When the conditions are brought about, we have a like change occurring in the physical characteristics of the tartar, which now assimilates more to the white variety. The yellowish brown variety generally accumalates in larger quantities about the teeth than the other. The white is most frequently found in the mouths of those of mucous temperaments, but is also, liable to be changed or modified by accidental causes.

Children in health have generally but little tartar on their teeth. The elements necessary for its production appears to be appropriated to the osseous structure, which at this period it may be inferred, require all such material for its perfection. M. Delabarre remarks on this subject, that "adults have more of it, especially after having passed their thirtieth year and advance toward old age. It is in the last periods of life that the mouth presents the largest quantity of it, because then the calcareous earth that abounds in our humors, becoming superfluous, exudes by means of the exhalents from the mucous membrane and skin." We suppose, however, that the salivary secretions may just as well, at this period of life, throw off this superfluity of calcareous earth as well as the mucus membrane, while the latter throwing off its excess of mucus becomes, as Dr. Harris remarks, "agglutinated" with the deposition taking place from the saliva in the fomation of the salivary calculus.

In our answer to this question we have thought it not necessary to allude to the effects specially of this substance on the teeth and gums, and the different effects resulting from different temperaments. The change, we may remark in this particular, is as great as that which is presented in the general physical characteristics of the tartar itself.

